# Comparison of the Core Training and Mobility Training Effects on Basketball Athletic Performance in Young Players: A Comparative Experimental Study

**DOI:** 10.3390/sports13110398

**Published:** 2025-11-06

**Authors:** Alessandra Amato, Cristina Cortis, Matteo Tropea, Marco Politi, Andrea Fusco, Giuseppe Musumeci

**Affiliations:** 1 Department of Biomedical and Biotechnological Sciences, Section of Anatomy, Histology and Movement Science, School of Medicine, University of Catania, Via S. Sofia n°97, 95123 Catania, Italy; alessandra.amato@unict.it (A.A.); matteotp@gmail.com (M.T.); m.politi98@gmail.com (M.P.); g.musumeci@unict.it (G.M.); 2 Department of Human Sciences, Society and Health, University of Cassino and Lazio Meridionale, 03043 Cassino, Italy; c.cortis@unicas.it; 3 European University of Technology EUt+, 03043 Cassino, Italy; 4 Department of Medicine and Aging Sciences, University “G. d’Annunzio” of Chieti-Pescara, 66100 Chieti, Italy; 5 Research Center on Motor Activities (CRAM), University of Catania, Via S. Sofia n°97, 95123 Catania, Italy

**Keywords:** team sport, performance, youth sport, neuromuscular control, balance, range of motion

## Abstract

This study compared the effects of core (CTG) or mobility training (MTG) on basketball-specific skills in youth players, focusing on dynamic balance. Both training modalities have a recognized role in enhancing performance, but few studies have examined their impact on this population. Thirty-one young (age 14.71 ± 2.27 years) males were assigned to an 8-week CTG or MTG. Overhead Squat, Y-Balance Test, Agility T-Test, Sit-and-Reach, Functional Hop Tests, and the Balance Error Scoring System were assessed before (pre) and after (post) the intervention for both dominant (D) and non-dominant (ND) limbs. Both groups improved the postero-lateral direction of the Y-Balance Test for the D (CTG, MD [95% CIs] = −8.108 [−15.620, −0.595], *p* = 0.035; MTG, MD [95% CIs] = −15.234 [−23.512, −6.956], *p* = 0.024) and ND (CTG, MD [95% CIs] = −9.110 [−16.150, −2.070], *p* = 0.013; MTG MD [95% CIs] = −13.899 [−21.657, −6.141], *p* = 0.001) limb and the medial reach for D (CTG, MD [95% CIs] = −17.279 [−26.364, −8.194], *p* = 0.001; MTG, MD [95% CIs] = −22.050 [−32.061, −12.039], *p* = 0.03) and ND (CTG, MD [95% CIs] = −9.309 [−17.093, −1.526], *p* = 0.021; MTG, MD [95% CIs] = −13.614 [−22.190, −5.037], *p* = 0.003), the Overhead Squat Test (CTG, MD [95% CIs] = −3.059 [−3.797, −2.321], *p* = 0.001; MTG, MD [95% CIs] = −3.643 [−4.456, −2.830], *p* = 0.001), and Agility T-Test (CTG, MD [95% CIs] = 0.572 [0.072, 1.073], *p* = 0.026; MTG, MD [95% CIs] = 0.696 [0.145, 1.248], *p* = 0.024) skills. Only CTG showed a significant improvement (MD [95% CIs] = −8.294 [−16.162, −0.426], *p* = 0.04) in single-leg hop performance for the ND limb. No significant improvements were observed in static balance or flexibility. No time × group effect was found. Both interventions improved key basketball-specific motor abilities and could be added to the basketball training session without adverse effect.

## 1. Introduction

Basketball is a high-intensity, intermittent team sport characterized by frequent accelerations and decelerations, rapid changes in direction, and vertical jumps that impose specific physical demands on athletes, requiring speed, power, and endurance to perform effectively [1]. Advancements in training strategies and modifications to the game’s rules have contributed to an increasingly fast-paced and physically demanding sport. Consequently, optimal physical conditioning, including good mobility, balance, agility, and, in general, the development of good neuromotor function [2], has become a key factor for basketball players, not only for maximizing performance but also for maintaining physical integrity and reducing injury risk. In response, physical conditioning programs have evolved to incorporate not only traditional sport-specific skills and general strength and endurance training, but also functional components designed to enhance these key motor abilities required for the game. Among these, core and mobility training have garnered increased attention in recent literature for their potential to both improve athletic performance and mitigate injury risk [3,4,5,6,7,8,9].

The core, which comprises the lumbopelvic–hip complex, functions as a central conduit for force transmission between the upper and lower extremities. In basketball, many movements originate in the lower limbs and culminate in upper-body execution (e.g., shooting, passing, rebounding). Sasaki et al. [10] examined the effect of core training in drop landings and single-leg squats in basketball players, reporting partial improvements in both. Hessam et al. [4] demonstrated that a 12-week core training program led to significant improvements in agility and dynamic balance in adolescent basketball players, directly linking core stability to these performances.

Mobility training, frequently mischaracterized as solely preventative, is increasingly recognized as a critical component of performance optimization. Mobility is not only related to joint range of motion but also gives information about neuromuscular control throughout that range under dynamic conditions [11]. Cook and colleagues demonstrated that exercises such as the deep squat are a predictive tool for evaluating the functional mobility of the hips, ankles, and shoulders, while also assessing motor control and core stability [12]. Limited joint mobility, particularly in the hips, shoulders, and ankles, has been associated with dysfunctional movement patterns, elevated injury risk, and decreased power output [13]. Enhancing mobility facilitates access to biomechanically advantageous positions, improves movement efficiency, and supports the execution of sport-specific skills such as the defensive stance or triple-threat position [14]. Mobility training, particularly focusing on the hips, has been shown to directly enhance functional range of motion and transfer to improvements in agility and sprint times in high school basketball players [15]. On the other hand, there are currently no studies linking hip mobility training with improved dynamic balance in young basketball players.

Therefore, the available evidence suggests that core training seems primarily to enhance the stability and efficiency of the kinetic chain, which is essential for powerful, coordinated movements such as jumping, cutting, and absorbing contact. In contrast, mobility training focuses on optimizing the range of motion and neuromuscular control around joints, which is a prerequisite for achieving optimal biomechanical positions and preventing movement restrictions that can lead to inefficiency or injury. However, a direct comparison of the effects of these two training methods on the same motor skill, assessed with standardized tests, is lacking. Addressing this gap is essential to determine whether each method has a specific efficacy, as they appear to target different yet complementary aspects of basketball performance. Such a comparison would allow for the optimal integration of each methodology, in terms of timing and objectives, alongside traditional sport-specific skill training. In basketball players, deficits in balance are a common risk factor for lower limb injuries [16]. For these reasons, the primary aim of our study will be to analyze the effect of the two training modalities on dynamic balance as assessed by a standardized test. Our secondary objective will be to compare any differences and simultaneous effects of the two modalities on the basketball-specific skills, agility, mobility, and flexibility.

By integrating these protocols into standard youth basketball practice, this study seeks to elucidate the most efficient training strategy for optimizing key physical qualities. The rationale for this comparison is not to determine a superior training modality per se, but to investigate whether core and mobility training elicit distinct, sport-specific adaptations. It is hypothesized that core training will preferentially enhance agility and dynamic balance by improving trunk stabilization and force transfer during rapid directional changes. In contrast, mobility training is expected to yield greater improvements in functional movement patterns by addressing joint kinematics and neuromuscular control, which are critical for achieving the deep stances and full ranges of motion required in basketball. The intervention protocols and the test battery proposed in this study have been specifically designed to reflect this rationale.

## 2. Materials and Methods

### 2.1. Participants

We recruited 36 basketball players belonging to the youth categories of the “Sporting Club Adrano A.S.D” basketball team (Catania, Italy). All the participants followed the same weekly basketball training routine, training three days per week for 90 min per session, for a total weekly basketball training volume of 270 min.

Individuals were eligible if they were without lesions or trauma to bones, ligaments, tendons, or muscles of the lower extremities in the previous 6 months, had no prior diagnosis of orthopedic disorders, and with at least 1 year of basketball experience. Participants were excluded if they did not meet the inclusion criteria, if they had stopped practicing basketball, or if they were regularly engaged in other sports disciplines. The study was conducted following the Declaration of Helsinki and was approved by the Scientific Committee of the ‘Research Center on Motor Activities’ at the University of Catania (Catania, Italy), protocol CRAM-57-2024, date of approval 17 July 2024. Before participation, all athletes read an informed consent form for the anonymous use of their data, which was then signed by their legal guardians and the adolescents themselves to participate in the study. They also completed a self-reported questionnaire, which served to confirm their eligibility and to gather personal information, lifestyle details, and information about their training habits. The “Waterloo Footedness Questionnaire” [17] was administered to identify the dominant (D) and non-dominant (ND) leg.

### 2.2. Study Design

Athletes were randomly distributed to two homogeneous groups: the core training group (CTG) (*n* = 18) or the Mobility Training Group (MTG) (*n* = 18). CTG conducted exercises that included concentric, eccentric, and isometric contractions targeting both deep and superficial core muscles. For the MTG, the protocol included static and dynamic stretching and targeted mobility exercises for key joints involved in basketball. We used block randomization [18], which provided a better guarantee that both groups would be of nearly equal size and have a similar age distribution. We defined the blocks according to three categories: U13, U15, and U19. Before participant enrollment, the primary investigator (A.A.) determined the sequence of random assignment to the two intervention groups (mobility training or core training) for the three blocks by using the Excel formula “=RANDOM ()”. This allowed us to maintain the allocation concealment. Then, a research assistant (M.T.) randomly drew the names of the participants from a pool and assigned them sequentially to the pre-established group sequence. This ensured that the researchers involved in enrolling participants could not know the upcoming group assignment, thus preventing selection bias.

All participants completed a test battery that identified tests suitable for analyzing the specific skills for basketball performance. The outcome assessors, M.P., together with a research assistant, conducted the physical performance tests and were blinded to the group assignment of the participants. M.P. and the research assistant were not involved in the training interventions. Participants were also instructed not to disclose their training group affiliation to the assessors.

The battery test (adapted from Read et al. [19] and Bird et al. [20]) was performed before (T0) and after (T1) 8 weeks of training and included seven tests, covering the mobility, agility, and static and dynamic balance skills. The tests included the Overhead Squat Test (OST), the Back Scratch Test, Sit-and-Reach Test (SRT), Agility T-Test, Y-Balance Test (YBT), Functional Hop Test, and Balance Error Scoring System (BESS).

### 2.3. Mobility, Balance, and Agility Assessment

#### 2.3.1. Overhead Squat Test

OST is a validated test included in the “Functional Movement Screen” that gives important information about the specific mobility of the basketball player and any motor dysfunction of the hips, knees, and ankles [19,21,22]. The specific scoring protocol used in this study is adapted from the original description by Cook et al. [12], and subsequent validation work by Minick et al. [23]. Participants had to perform eight consecutive squats with hands at the level of the iliac crests and two squats with arms extended above the head with feet not exceeding shoulder width. The operator was instructed to observe the behavior of the ankle joint, knee joint, and pelvis. A poor ability to maintain pelvic anteversion during movement, visibly impaired degrees of bending of the ankle and knee joints, and lateral movements of the latter (stance in valgus or varus). If none of these issues were present, each joint received a score of one. This phase of the test, performed with the hands placed on the hips, provided a maximum of 3 points available (one point for each of the three areas assessed: ankles, knees, and pelvis).

After one minute, the test was repeated with the arms extended above the head to assess the mobility of the shoulder joint and ankle dorsiflexion under closed kinetic chain conditions. In case the athlete’s ear was visible during the squat, this meant poor mobility of this joint, so a score of zero was assigned; otherwise, a score of one was assigned. Ankle dorsiflexion was evaluated by observing whether the tibia remained parallel to the torso in the sagittal plane. If not, the score was zero; otherwise, the score was one. This second phase evaluated both left and right shoulders and ankles, for a maximum of 4 points. Finally, the scores from all 7 criteria (3 from the first phase and 4 from the second) were summed to obtain the total OST score. A higher score reflected better movement quality and control during the squat.

#### 2.3.2. Back Scratch Test

The Back Scratch Test was developed and validated to evaluate upper-body flexibility [24]. We measured the total shoulder range of motion by recording the distance (negative value) or overlap (positive value) in cm between the middle fingers as they reached behind the back. The participants performed the test twice with each arm, alternating sides, with no recovery time between right and left limbs; the final score in cm was considered the best attempt from both arms. The operator measured laterally to the hand that was above, between the two extremities of the middle fingers, when there was an overlap between hands [25].

#### 2.3.3. Sit-and-Reach Test

The SRT is a reliable and validated method for assessing the flexibility of the back muscle chain [26]. The assessment is carried out using a standardized box (Baseline 12-1085, Fabrication Enterprises, New York, NY, USA). Participants sat on the floor with their legs fully extended and the soles of their feet placed flat against the front of the box. From this starting position, participants were instructed to inhale, then exhale while bending forward at the waist with arms and fingers extended, reaching as far as possible along the centimeter scale positioned on top of the box. Three repetitions were performed, and the average of the three attempts was considered for data analysis. There was no recovery time between sets, as only the time needed to slowly return to the starting position was allowed. The effectiveness of doing three repetitions to obtain a reliable result in SRT has been previously demonstrated [27].

#### 2.3.4. Agility T-Test

The most commonly used test to assess agility is the Agility T-Test. According to the protocol, originally described by Semenick [28] and validated for reliability and validity by Pauole et al. [29], participants were required to sprint forward from a starting line, touch the first cone 10 m in front of the starting line with the right hand, shuffle left 5 m and touch the second cone with the left hand, shuffle right 10 m and touch the third cone with the right hand, shuffle back 5 m to the first cone, then backpedal 10 m as fast as possible to the start line [30]. First, the test administrator explained and performed the test slowly. Then, the participants performed a familiarization repetition of the test, followed directly by a single test at maximum intensity, during which the time (in seconds) was recorded.

#### 2.3.5. Balance Error Scoring System

The BESS is designed to assess an individual’s ability to maintain static balance. The protocol and error scoring criteria used were based on the original development by Riemann et al. [31] and the subsequent widespread application and review by Bell et al. [32]. Participants had to maintain six conditions: double-leg stance, single-leg stance (on the ND leg), and tandem stance (ND foot behind the D one), for 20 s with eyes closed on both a firm and foam surface. A 20 s rest was provided between conditions. One error point was recorded for: opening the eyes; removing hands from the hips, stepping, stumbling, or falling; lifting the forefoot or heel; moving the hip into more than 30° of abduction; or remaining out of the test position for more than 5 s. The final BESS scores were the sum of errors for all conditions.

#### 2.3.6. Y-Balance Test

The YBT is a valuable musculoskeletal screening tool to assess dynamic balance and the identification of lower limb and trunk movement dysfunction in basketball players [33]. Plisky et al. demonstrated that diminished performance on the YBT is associated with an increased risk of non-contact lower extremity injuries [34]. Its validity in basketball is further supported by its close approximation of the split-stance posture, a common and functional biomechanical position frequently adopted during gameplay.

We followed the methodology of Fullam et al. [35] and the original, foundational protocol described by Plisky et al. (2006) [36]. We constructed a “Y” with tape on the floor, one oriented anteriorly and the other two in the posteromedial and posterolateral directions, both at a 135-degree angle to the first. The three arms of the “Y” were then calibrated with the tape measure to simplify the collection of results. The athlete stood in the center of this “Y,” with his toe resting at the point of convergence of the three pieces of tape, barefoot and with his hands on his hips. The goal was to move a reach block as far as possible with the non-supporting foot along the anterior directions (ANT), posteromedial directions (PM), and posterolateral directions (PL), without crushing it or putting pressure on the floor, without performing a push on the reach block to make it go farther, and return with the non-supporting foot to the supporting foot without touching the floor. The distance traveled by the reach block was then measured with a meter from the point of convergence of the arms of the “Y” to the nearest edge of the reach block in each direction and for both D and ND limbs. Therefore, the six derived measures (D ANT, D PL, D PM, ND ANT, ND PL, and ND M) in cm were considered separately for data analysis. To reduce confounding by anthropometry, normalized reach distances were calculated for each measure using the following formula: Reach distance÷Limb length×100. The YBT protocol and normalization procedure have demonstrated excellent inter-rater and test–retest reliability [34]. Participants completed a single familiarization trial before the test for both the D and ND limbs.

#### 2.3.7. Functional Hop Tests

The Functional Hop Tests are a validated battery test [37] that offers valuable insight into lower limb strength, explosive power, and dynamic stability [38,39]. The specific procedures were implemented as per the original reliability studies by Ross et al. [37] and the normative data collection in basketball players by Myers et al. [39]. The test was conducted in 4 stages:

1. Single-Leg Hop for Distance: The athlete begins on the testing leg with his toe at the starting line and performs a forward hop, landing on the same leg. The distance is measured from the starting line to where the heel lands. The athletes were instructed to stabilize the landing with good control and minimal movement for at least three seconds.
2. Triple Hop for Distance: Starting on the testing leg with the toe at the start line, the athlete performs three powerful forward hops using the same leg. The total distance was measured from the start to the heel’s landing point after the third hop. Athletes were instructed to maintain balance and hold the final landing position steadily for a minimum of three seconds.
3. Crossover Triple Hop for Distance: The athlete stands on the same side of a centerline tape as the leg being tested (e.g., right side for the right leg), with the toe at the start line. Each participant performs three continuous hops forward, crossing over the tape in a zig-zag pattern with each hop. The total distance was measured to the heel’s landing after the third hop. The athlete was instructed to hold the final position with control and limited sway for at least three seconds.
4. Six-Meter Timed Hop on One Leg: The athlete started on the testing leg with the toe on the start line. On the command “3, 2, 1, go,” he was instructed to hop as quickly as possible over a six-meter distance using only that leg. The timer was stopped when the athlete crossed the six-meter finish line.

Participants performed one submaximal trial for each stage to familiarize themselves with the task. Then, they performed three maximal trials for both D and ND limbs for each of the four stages. The best hop distance of the three trials for the first, second, and third stages was used for statistical analysis [39,40]. Participants had 30 s of rest between the trials of the same stage and one minute of rest between stages [41].

#### 2.3.8. Training Programs

The exercise interventions are described in accordance with the Consensus on Exercise Reporting Template (CERT) guidelines [42] to enhance the clarity, reproducibility, and completeness of the intervention reporting. Participants performed the training session under the supervision of an Italian basketball federation coach (M.T.), who also collected information on adherence to exercise by calling the roll of all players involved in the study at each session. Additionally, during the session, M.T. was required to record any adverse events reported by participants, including the onset of pain or fatigue before, during, or after the protocol. Both groups trained three times per week for 8 weeks before their ordinary basketball training session for a total of 24 sessions.

#### 2.3.9. Core Training Protocol

The protocol was adapted from Sasaki et al. [10] and consisted of three exercises: plank, side plank, and Nordic hamstrings exercise (NHEs). Planks and side planks were performed for two sets of 30 s, while the NHE was performed once for progressively 3 to 15 repetitions according to the protocol [10]. The recovery time between sets and between exercises was 30 s to maintain the basketball game’s physiological demands of a 1:1 work-rest ratio of [43].

**Figure 1:**
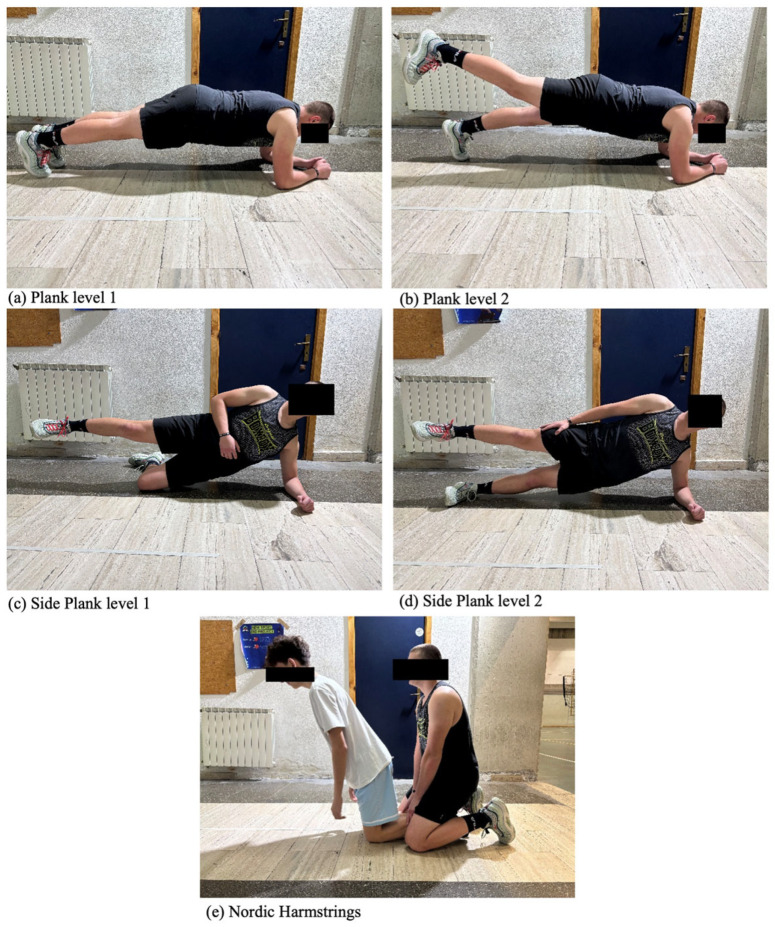
Core training exercises. The figure shows pictures of the Participant performing each exercise of the core training protocol: (**a**) Plank level 1; (**b**) plank level 2; (**c**) side plank level 1; (**d**) side plank level 2; (**e**) Nordic hamstring.

The plank was performed by resting the forearms and toes on the ground, keeping the pelvis tilted backward to increase the work of the abdominal muscles. The exercise consisted of two sets of thirty seconds each (Figure 1a). From week 5 to week 8, the difficulty level of the exercise increased, and the participant had to lift a limb and hold the position throughout the series (30 s). In the first series, one limb was kept lifted, and in the next series, the contralateral limb was kept lifted (Figure 1b). The side plank was performed by lying on the side on the ground, keeping the head, shoulders, torso, pelvis, and lower limbs aligned. The forearm was placed on the side on which the athlete rested, and the leg of the same side on the ground; then, a ninety-degree bend at knee level was performed. In addition, the hip closest to the ground was lifted to keep the obliques and transverse muscles in tension. The lower limb farthest from the ground was kept raised off the ground, in line with the rest of the body (Figure 1c). After the first 4 weeks, to increase the level of difficulty, the exercise was performed by stretching the lower limb resting on the ground (Figure 1d). The exercise consisted of two sets of thirty seconds per side. Plank exercises were included to target the anterior core stabilizers, critical for maintaining spinal alignment and facilitating force transfer during athletic movements [44]. To perform the NHE, athletes started from a kneeling position, with the support of a partner sitting on the athlete’s legs, and they had to let themselves fall forward, slowing down the fall as much as possible and keeping the torso and pelvis vertical (Figure 1e). The NHE was included as a key component of the core training program, being an effective hamstring strengthener and putting high demand on lumbopelvic stabilizers. Electromyographic studies confirmed that the NHE elicits moderate-to-high activation of the erector spinae and internal oblique muscles, which are essential for maintaining a neutral spine and stable pelvis against the gravitational and inertial forces during the eccentric lowering phase [44,45,46], critical for the transfer of forces across the kinetic chain. We assumed that the NHE would enhance core stability under dynamic, high-load conditions, with a direct transfer to the functional outcomes assessed in this study. Specifically, improved lumbopelvic control is a known determinant of performance in dynamic balance tasks like the YBT [47] and contributes to more efficient force transfer during the deceleration and re-acceleration phases of agility and hopping tasks [48,49]. Therefore, the NHE was selected to improve the integrated stability and function of the posterior chain, which is fundamental to basketball performance. The repetitions ranged from three to five for the first level, five to ten for the second level, and ten to fifteen for the third level (reached progressively over the 8 weeks). The exercise was performed in a single set.

#### 2.3.10. Mobility Training

The protocol, including a 30 s rest between each exercise, was adapted from Antoranz et al. [15].

**Figure 2:**
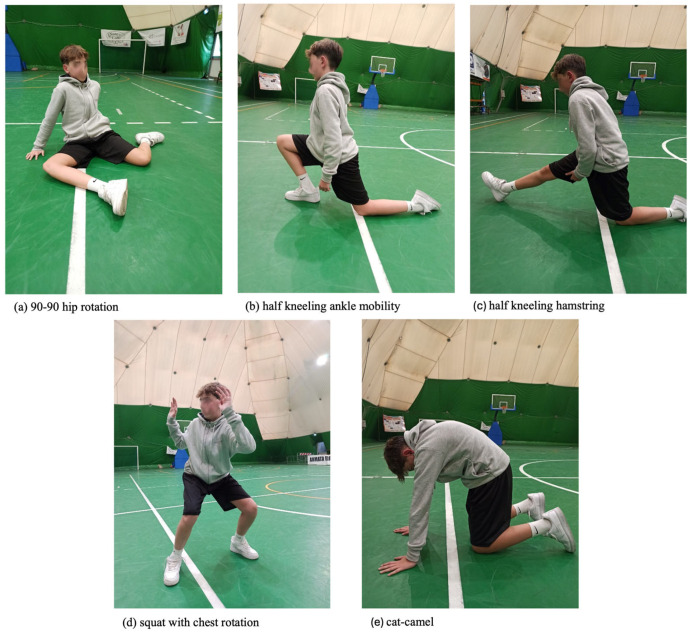
Mobility training exercises. The figure shows pictures of the Participant performing each exercise of the Mobility training protocol: (**a**) 90–90 hip rotation; (**b**) half-kneeling ankle mobility; (**c**) half-kneeling hamstring; (**d**) squat with chest rotation; (**e**) cat-camel.

The first exercise was the 1 min 90–90 hip rotation, focusing on stretching the hip flexors and increasing external hip rotation. The athletes were sitting on the floor with their legs in a 90–90 position. They gently rotated their hips outward, thus bringing their knees toward the ground (Figure 2a). The exercise lasted one minute for one repetition.

The second exercise was the half-kneeling ankle mobility. Athletes started from the lunge position with the knee on the ground, and they brought the front knee forward to induce a dorsal flexion of the tibio-tarsal joint. Athletes had to maintain the position for 30 s per leg for one repetition (Figure 2b). No recovery was allowed between left and right limb repetition. This drill targets ankle dorsiflexion, which is a known limiting factor in Overhead Squat performance and dynamic balance [50].

The third exercise was the half-kneeling hamstring stretch. From the starting position of the previous exercise, athletes extended the leg of the front knee forward to generate a stretch of the hamstrings, maintaining a 90 degree angle between the trunk and the extended leg. When they reached the maximum stretch phase, they kept it for 2 s, and then they returned to the starting position. The exercise lasted 30 s for each leg and was repeated once (Figure 2c).

The fourth exercise was the squat with chest rotation. The athletes performed a squat starting from a standing position with the shoulders abducted at 90° and the elbows also flexed at 90°. At the maximum squat phase, they twisted their torso in both directions, ensuring that the pelvis does not rotate towards the side of the twist (Figure 2d). A set of 12 repetitions was performed on each side. The last exercise was the cat-camel. The athletes started in a quadruped position, with hands at shoulder height and knees at hip height. From this position, creating an anteversion of the pelvis, they brought the abdominal area towards the ground, keeping the shoulders and knees fixed. After holding the position for two seconds, they did the opposite, arching their back toward the ceiling, thus causing a retroversion of the pelvis. The exercise was repeated once for one minute (Figure 2e).

### 2.4. Load Progression and Session Structure

To ensure a progressive and comparable internal load to the CTG, the progression criteria for the mobility exercises were based on the controlled increase in the range of motion (ROM) and movement quality. For the static exercises (90–90 hip rotation and half-kneeling ankle mobilization), participants were instructed to gradually increase the stretching intensity week by week, aiming for mild discomfort without pain. For the dynamic exercises (half-kneeling hamstring stretch, squat with thoracic rotation, and cat-camel), the progression focused on achieving a greater ROM and superior motor control with each repetition, while the execution time and repetition count remained fixed. Both training protocols were designed to be matched for total time under tension. Excluding one exercise per protocol where repetitions were the metric (NHE for core training; squat with thoracic rotation for mobility training), both sessions had an identical total duration of 6 min and used consistent 30 s rest intervals between sets and exercises.

### 2.5. Statistical Analysis

A priori power analysis was conducted using G*Power software (G*Power, 2007, version 3.1, Düsseldorf, Germany) to estimate the required sample size for a repeated measures ANOVA with a mixed design. Based on effect sizes observed in the previous literature involving similar athletic populations and interventions, a medium effect size (f = 0.25) was selected. With an alpha level of 0.05, power set at 0.80, and an assumed correlation of 0.5 between repeated measures, the analysis indicated that a minimum total sample size of 34 participants (17 per group) would be required to detect a statistically significant interaction effect. The analysis used two primary statistical approaches: Linear Mixed-Effects Models (LMMs) with random intercepts for participants, and traditional repeated-measures Analysis of Variance (ANOVA) (2 conditions × 2-time intervals) used to analyze the differences within and between MTG and CTG for each dependent variable. The LMM was estimated via maximum likelihood with robust (clustered) standard errors, serving as a sensitivity analysis to confirm the robustness of the ANOVA findings. The significance threshold was set at α = 0.05 (two-tailed). To address multiple comparisons, False Discovery Rate (FDR) correction was applied within the YBT (six directions) and Functional Hop Test (eight measures) families. The normality of the data was checked by the Shapiro–Wilk test. An independent *t*-test was conducted to assess baseline differences among the participants’ characteristics between the groups. Post hoc pairwise comparisons were conducted with a Bonferroni adjustment to control for Type I error. The assumption of homogeneity of variance was assessed using Levene’s Test for each variable at each time point. Effect sizes for within-group changes over time are reported as Hedges’ g_av with 95% confidence intervals, calculated using a standard formula to correct for small sample bias. Between-group effect sizes were reported as Cohen’s d with their 95% confidence intervals for all outcome measures. The SPSS^®^ (IBM^®^, Chicago, IL, USA) version 29.0.2.0 software and the Jamovi (jamovi project, 2022, Version 2.3, Sydney, Australia) software were used for statistical analysis.

## 3. Results

**Figure 3:**
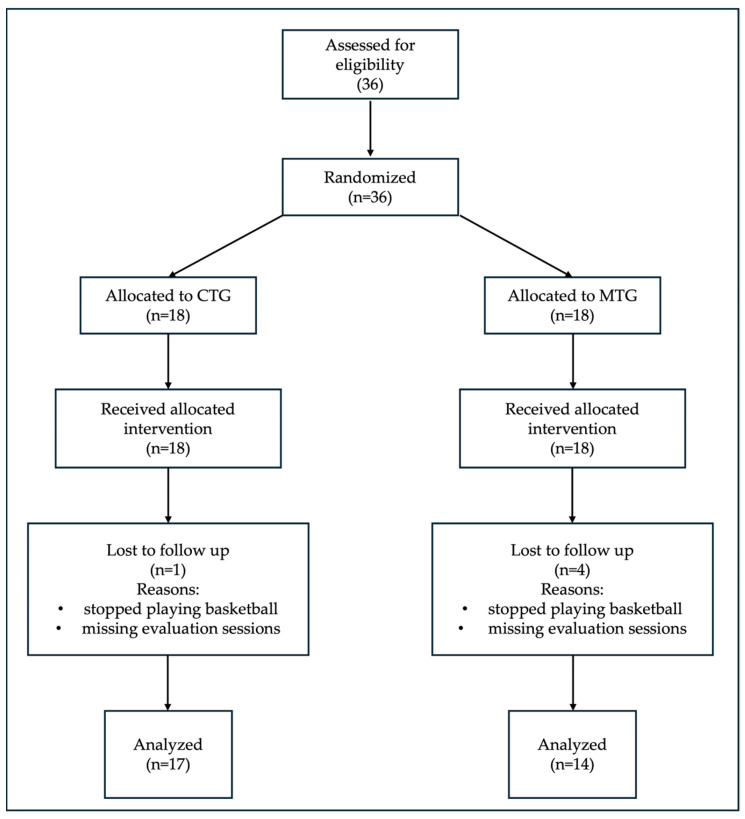
CONSORT flow diagram of the study. The diagram shows participant recruitment, allocation, and analysis through the phases of the randomized trial. Control group (CTG); mobility group (MTG). Thirty-six male basketball players were randomized into two intervention groups. Five participants were lost to follow-up due to discontinuation of basketball practice and non-participation in evaluation sessions (partially at T0 and completely at T1). Thirty-one participants were included in the final data analysis.

Five participants were excluded from data analysis because they had stopped practicing basketball and did not participate in the evaluation sessions. Thirty-one participants (age: 14.8 ± 2.04 years, weight: 70 ± 11.8 kg, BMI: 22.9 ± 3.07 kg·m^−2^) completed both the T0 and T1 evaluations and were included in the data analysis, comprising CTG (*n* = 17) and MTG (*n* = 14). The overall mean training sessions adherence was 86.96 ± 10.02%. Table S1 (Supplementary Materials) reports adherence for each participant as the percentage of sessions attended. The participant flow through the study is summarized in the CONSORT diagram (Figure 3).

The independent *t*-test showed no significant differences between groups at baseline (*p* > 0.05). Table 1 summarizes baseline participants’ characteristics per group.

A violation was found for T0 posterior lateral YBT for the D (*p* = 0.018) and ND limb YBT anterior (*p* = 0.042). For all other variables, the assumption was met (all *p* > 0.05). Table S2 in the Supplementary Materials shows Levene’s Test of equality of error variances for all outcome measures.

### 3.1. Main Effect Analysis

Mixed ANOVA showed a significant main effect of time, for the OST F (1, 29) = 155.86, *p* < 0.001, partial η^2^ = 0.843 (very large), the Agility T-test F (1, 29) = 12.13, *p* = 0.002, partial η^2^ = 0.295 (large), the YBT ND PL F (1, 29) = 20.18, *p* < 0.001, partial η^2^ = 0.410 (large), the YBT ND PM F (1, 29) = 16.39, *p* < 0.001, partial η^2^ = 0.361 (large), the YBT D PM F (1, 29) = 35.40, *p* = 0.001, partial η^2^ = 0.550 (large), and the YBT D PL F (1, 29) = 18.24, *p* < 0.001, partial η^2^ = 0.386 (large), the ND Hop Test 6 m F (1, 29) = 4.53, *p* =0.042, partial η^2^ = 0.135. The main effect of group was significant, only for the OST F (1, 29) = 4.36, *p* = 0.046, partial η^2^ = 0.131 (medium-to-large). The time × group interaction was not significant for any of the variables considered. Table S3 (Supplementary Materials) presents the F-value, degrees of freedom, *p*-value, and partial η^2^ for the main effect for each variable from ANOVA analysis.

LMM showed a significant main effect of time in the YBT PL for both the D limb (F (1, 58) = 14.203, *p* < 0.001) and the ND limb (F (1, 58) = 16.843, *p* < 0.001), without a significant group effect or group × time interaction being observed. A significant main effect of time was also found for YBT in the PM direction. This was true for the D limb (F (1, 58) = 6.557, *p* = 0.013) and the ND limb (F (1, 58) = 10.691, *p* = 0.002). For the OST total score, significant main effects were found for both group (F (1, 58) = 7.001, *p* = 0.010) and time (F (1, 58) = 90.900, *p* < 0.001).

No significant effects of group (*p* = 0.496) or time (*p* = 0.109) were found for the Agility T-Test performance. For both the D and ND limbs, the analyses of single-leg hop distance showed no significant main effects for Group or Time (all *p* > 0.05). No significant main effects of group or time were found for the triple hop distance on either limb (all *p* > 0.05). No significant effects of group or time were found for the crossover triple hop performance on either the D or ND limbs (all *p* > 0.05). The time to complete the six-meter hop test showed no significant changes for either group or time on both the D and ND limbs (all *p* > 0.05). The time × group interaction was not significant for any of the variables considered. Table S4 compares the main effect of time across statistical methods.

### 3.2. Within-Group Pairwise Comparison

#### Mixed ANOVA

The within-group changes, effect sizes, and between-group differences in change are detailed in Table S5 (Supplementary Materials). In addition to significant time effects, both groups demonstrated substantial absolute and relative improvements in key performance metrics.

Within-group analysis revealed a statistically significant improvement in the squat test for both CTG (MD [95% CIs] = −3.059 [−3.797, −2.321]; *p* = 0.001) and MTG (MD [95% CIs]= −3.643 [−4.456, −2.830]; *p* = 0.001). These changes corresponded to large relative improvements of 115.5% and 254.9%, respectively, with very large effect sizes (Hedges’ g > 2.8), indicating a robust intervention effect. In the CTG, significant gains were also observed in the ND Hop Single-Leg Test (MD [95% CIs] = −8.294 [−16.162, −0.426]; *p* = 0.040), representing a 7.2% improvement, which was greater than the non-significant 1.2% change observed in the MTG. The between-group difference in change for this variable was −6.94 cm [95% CI: −17.69, 3.82]. To investigate the effects of the interventions on functional lower-limb asymmetry, we calculated the Limb Symmetry Index (LSI) for the single-leg hop test (ND Hop Single-Leg ÷ D Hop Single-Leg × 100), and we performed a mixed ANOVA on LSI. An LSI of 100% indicates perfect symmetry. At baseline, there was no significant difference in LSI between groups (*p* = 0.674). A mixed-model ANOVA revealed no significant time × group interaction (F (1, 29) = 0.036, *p* = 0.851), indicating that the changes in symmetry over time did not differ significantly between the CTG and MTG. The main effect of time was also non-significant (F (1, 29) = 0.298, *p* = 0.589). Descriptively, the mean LSI increased by 1.95% in the CTG (from 101.16% to 103.11%) and by 0.95% in the MTG (from 103.30% to 104.25%).

Furthermore, the number of athletes with a clinically relevant asymmetry (LSI < 90%) in the CTG was reduced from 4/17 at T0 to 2/17 at T1. No such reduction was observed in the MTG (3/14 at both T0 and T1).

For dynamic balance, CTG improved ND YBT PM (MD [95% CIs] = −9.309 [−17.093, −1.526]; *p* = 0.021) and PL reaches (MD [95% CIs] = −9.110 [−16.150, −2.070]; *p* = 0.013), D YBT PM (MD [95% CIs] = −17.279 [−26.364, −8.194]; *p* = 0.001) and PL reaches (MD [95% CIs] = −8.108 [−15.620, −0.595]; *p* = 0.035). Similarly, the MTG showed significant improvements in ND YBT posteromedial (MD [95% CIs] = −13.614 [−22.190, −5.037]; *p* = 0.003) and PL reaches (MD [95% CIs] = −13.899 [−21.657, −6.141]; *p* = 0.001), D PL reaches (MD [95% CIs] = −15.234 [−23.512, −6.956]; *p* = 0.02) and PM reach (MD [95% CIs] = −22.050 [−32.061, −12.039]; *p* = 0.03). The MTG consistently showed larger relative improvements across all these YBT directions (D PL: MTG +16.7% vs. CTG +8.5%). Both groups significantly improved in the Agility T-Test CTG (MD [95% CIs] = 0.572 [0.072, 1.073]; *p* = 0.026), MTG (MD [95% CIs] = 0.696 [0.145, 1.248]; *p* = 0.02). The magnitude of change was similar, with mean time reductions of 0.57 s (−4.0%) and 0.70 s (−4.8%) for the CTG and MTG, respectively. The between-group difference in change was minimal (−0.12 s [95% CI: −0.87, 0.62]), indicating comparable effects on agility.

### 3.3. Between-Group Pairwise Comparison

Between-group comparisons at T0 and T1 showed no statistically significant differences between CTG and MTG for most variables. However, the ANOVA mixed model showed a significant difference in the ND YBT posterolateral reach (MD [95% Cis = 8.019 [1.327, 14.712], *p* = 0.021; Cohen’s d = 0.66, 95% CI for d: −0.06 to 1.38) and squat test (MD [95% Cis = 1.218 [0.128, 2.309], *p* = 0.030; Cohen’s d = 0.63, 95% CI for d: −0.09 to 1.35) between groups at T0. However, since this difference was observed only at baseline, it is reasonable to assume that it was a random occurrence. Table S5 (Supplementary Materials) shows between-group comparisons (CTG vs. MTG) for all outcome measures at T0 and post-intervention T1 by mean difference (CTG—MTG) with standard error, *p*-value, and 95% confidence intervals (95% CIs). However, the analysis of between-group differences in change scores (Table S6, Supplementary Materials) revealed specific areas where the interventions may have diverged. While the time × group interaction in the ANOVA was not significant, the between-group difference in change for the Sit-and-Reach test was 2.72 cm [95% CI: 1.08, 4.36], indicating a significantly greater improvement in flexibility for the MTG. A similar, statistically significant difference in change was observed for the YBT ND ANT (4.65% [95% CI: 0.06, 9.24]), favoring the MTG.

Table 2 shows descriptive statistics (means and standard errors) for all outcome measures.

### 3.4. Sensitivity Analysis: Methodological Convergence Mixed ANOVA vs. LMM Comparison

**Figure 4:**
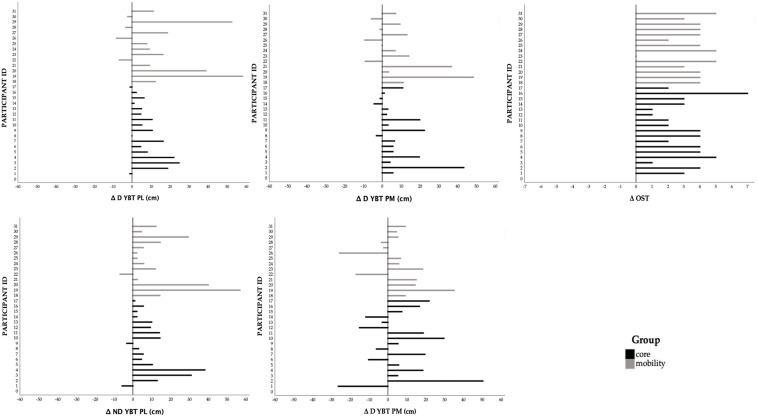
Individual variation (T0–T1) for Y-Balance Test (YBT) posterolateral reach (PL), posteromedial reach (PM) for dominant (D) and non-dominant (ND) limb, and Overhead Squat Test (OST) scores that showed statistically significant improvements. Lines represent change over time for each participant, stratified by intervention group.

The results demonstrated strong methodological convergence, identified significant time effects from T0 to T1 for YBT ND PM (mixed ANOVA: *p* < 0.001, LMM: *p* = 0.002), YBT ND PM (both *p* < 0.001), YBT D PM (mixed ANOVA: *p* = 0.001, LMM: *p* = 0.014), YBT D PL (both *p* < 0.001), and OST (LMM: *p* < 0.001; mixed ANOVA: *p* < 0.001). All methods agreed on the lack of significant time × group interactions (all *p* > 0.50). All approaches consistently found no improvements in hop tests, static balance, or flexibility. The Agility T-Test and the 6 m hop test showed methodological dependence, with significant improvement in ANOVA (*p* = 0.002) but not in LMM (*p* = 0.114). The 6 m timed hop approached was significant in ANOVA (*p* = 0.042) but not in LMM (*p* = 0.342) Figure 4 shows individual variation for the variable, resulting in statistically significant improvements in all methods.

## 4. Discussion

This study aimed to analyze the influence of core or mobility training programs on the development of specific physical abilities in young basketball players. The primary finding of this study, confirmed through LMM with hierarchical FDR correction, is that both training protocols elicited significant improvements in specific domains of dynamic balance in the PL and PM directions of the YBT, our primary outcomes, and functional mobility as measured by the OST (all pFDR < 0.05). Critically, the absence of significant time × group interactions across all statistical approaches provides robust evidence that the rate and magnitude of improvement did not differ between interventions. However, the analysis of the estimated changes and their variability provides deeper insights. CTG led to significant improvements in the Functional Hop Test single leg for the ND limb (7.2% vs. 1.2% in MTG). This finding did not survive FDR correction when considered within the broader context of the eight hop test measures (pFDR = 0.819). This suggests that what appeared to be a potential advantage in dynamic trunk control and lower-limb power transmission may represent a Type I error rather than a robust training effect. The hop test single leg is predictive for lower limb strength, explosive power, and dynamic stability [51]. A previous study showed improvement in an exercise with similar biomechanics to the hop test, the single-leg squat, after 8 weeks of core-muscle training, resulting in better neuromuscular control [10].

Core strengthening changes trunk kinematics in the coronal and sagittal planes, and sagittal-plane trunk control significantly influences knee biomechanics. Kulas et al. showed that hamstring activation and anterior shear at the knee are affected by trunk posture [48], while other authors observed that greater trunk flexion during landing and cutting is associated with increased hip and knee flexion angles [52,53]. Core stability training has been shown to improve single-leg hop performance and reduce asymmetry between limbs [54], and similar effects have been observed in the upper limb [55]. However, in Mohammadi’s study [54], symmetry was assessed between the injured and uninjured limb, with the performance of the injured limb approaching that of the uninjured one after the training period. We observed improvement only in the ND limb. It may be reasonable to draw a parallel between the disadvantaged condition of the limb and the similar gains reported in both studies in response to core stability training. The greater improvements in the ND limb’s reach further underscore the asymmetrical adaptations that can occur due to habitual dominance in limb use. ND limbs are often less efficient in neuromuscular control, making them more responsive to proprioceptive and mobility stimuli [56]. Therefore, incorporating exercises such as the plank, particularly those involving a single-leg position (Figure 1b,d), may have enhanced limb stability. However, the functional asymmetries calculated through the LSI revealed no significant time × group interaction. This indicates that both training protocols had similar effects on inter-limb symmetry over the 8 week intervention period. The CTG showed a slightly greater numerical improvement in symmetry (+1.95%) compared to the MTG (+0.95%). While this pattern aligns with the observed improvement in ND limb performance in the CTG, the lack of statistical significance suggests this was not a consistent effect across all athletes. The unilateral components of the core training protocol may provide a stimulus for addressing asymmetries, but our study might have lacked the statistical power to possibly detect such an effect. Future studies with larger samples specifically targeting athletes with pre-existing asymmetries are needed to clarify this potential relationship. The lack of clear differential effects between the two training modalities, despite both groups showing significant improvements, has already been observed in youth athletic populations [57,58]. Several factors may explain this finding. First, the intervention duration of 8 weeks might have been sufficient to elicit general neuromuscular adaptations but insufficient for more specific, modality-dependent adaptations to fully manifest. Second, the outcome measures used are global performance tests, and they may not be sensitive enough to detect differences in the neuromuscular mechanisms targeted by core versus mobility training. While core training might enhance trunk stiffness, and mobility training might improve joint kinematics, both pathways could lead to a similar enhancement in overall dynamic performance during a complex task like the YBT or Agility T-Test. The principle of training specificity suggests that transfer to sport-specific skills is complex, and the relationship between isolated physical qualities like core strength or mobility and integrated athletic performance is not always direct [49]. Finally, in adolescent populations, who are highly responsive to any structured training stimulus, the mere introduction of a novel, supervised training regimen can produce significant gains, potentially overshadowing more subtle inter-modal differences.

Significant OST improvements were observed in both groups and remained robust after multiple comparisons correction. The MTG demonstrated a remarkably large relative improvement of 254.9%, compared to 115.5% in the CTG, though the between-group difference in change was not significant (0.58 points [−0.54, 1.71]). This is particularly relevant in athletic populations, such as basketball players, where optimal joint mobility is critical for performance and injury prevention. These findings are consistent with previous literature suggesting that dynamic and targeted mobility exercises can enhance functional movement patterns [59]. Furthermore, the OST, often used as a screening tool for movement quality and functional deficits, showed improvements aligned with increased neuromuscular control and joint mobility. This test is especially sensitive to limitations in thoracic spine extension, hip and ankle dorsiflexion, and core stability, all of which are crucial for the explosive and multidirectional movements required in basketball [19,21,22]. Moreover, squatting ability is essential to the biomechanics of an effective defensive stance or triple-threat position in basketball. Several studies have emphasized the relationship between OST performance and functional athletic outcomes. Sever et al. (2023) [60] demonstrated that the overhead deep squat was one of the most informative components of the Functional Movement Screen tool in assessing global movement quality in athletes. Athletes with higher OST scores demonstrated better biomechanical efficiency during sport-specific tasks. Also, Tee and colleagues supported the use of the Overhead Squat as a diagnostic tool for identifying mobility restrictions and movement compensation patterns [61]. The observed improvements in OST performance following the interventions may be attributed to enhanced neuromuscular coordination, which promotes more efficient motor unit recruitment and synchronization. This neuromuscular adaptation not only contributes to greater movement economy but also reinforces joint stability, which is essential in mitigating the risk of common sports injuries such as anterior cruciate ligament tears and ankle sprains [62]. Proprioceptive input and sensorimotor integration are enhanced through dynamic mobility training, fostering improved postural control and reflexive stability during complex athletic tasks [63]. Therefore, our findings reinforce the growing body of evidence that both traditional strength-based protocols and mobility-oriented training can positively influence lower-limb muscular function, joint integrity, and movement control. YBT posteromedial and posterolateral directions performance improved significantly in both groups under investigation. Notably, the MTG showed consistently larger relative improvements across all YBT directions (e.g., D PL: MTG +16.7% vs. CTG +8.5%), and the individual response plots (Figure 4) confirmed a highly consistent positive trend across nearly all participants in both groups. The YBT is a validated clinical tool and has been widely employed in both athletic and rehabilitative settings to identify deficits and predict injury risk. Specifically, the posteromedial and posterolateral directions are considered more challenging due to the diagonal movement patterns that require greater motor control, core activation, and proprioceptive integration than the anterior direction [36] and were utilized to assess neuromuscular characteristics, including dynamic balance and flexibility [47]. The improvement observed in both groups may be attributable to the focus on joint range of motion and neuromuscular facilitation that such exercises typically emphasize. Enhanced joint mobility, particularly at the hip, ankle, and thoracic spine segments directly stimulated in our mobility exercises, has been associated with improved sensorimotor control, which is critical for maintaining balance during dynamic tasks [51]. Hip mobility plays a central role in allowing the body to shift weight efficiently during single-leg stance tasks, while ankle dorsiflexion is essential for effective forward progression and stabilization [50]. Moreover, mobility exercises may stimulate proprioceptive mechanisms by involving end-range joint positions and controlled oscillatory movements that challenge the somatosensory and vestibular systems. Studies have shown that proprioceptive enhancement through mobility-focused training can significantly improve dynamic balance and reduce the risk of falls or injury, both in athletic [64] and pathological populations [65], and rebounding workouts have also been reported to induce rapid balance adaptations [66]. Data suggests that mobility and core training, stimulating proprioceptive pathways, and engaging complex motor patterns, may confer a functional advantage in tasks demanding multidirectional balance control, particularly in posterolateral and posteromedial movements, such as the frequent split stance during a basketball game, optimizing dynamic postural control. The Agility T-Test also showed significant improvement in both groups (mean differences CTG: 0.57, MTG: 0.70), corresponding to relative improvements of −4.0% and −4.8%, respectively. The between-group difference in change was minimal (−0.12 s [−0.87, 0.62]), indicating identical effects on agility and suggesting that enhancements in core stability or joint mobility may translate into faster and more coordinated changes in direction. Given that agility is a composite skill involving strength, coordination, and balance, the improvements across both interventions reinforce the idea that such training modalities can benefit athletic movement efficiency. In addition, given that previous studies have identified agility, assessed via the *t*-test, as a reliable predictor of playing time in comparable populations, the inclusion of such protocols may directly influence athletes’ game performance [67,68]. Conversely, no significant changes were observed in any of the eight Functional Hop Test measures after FDR correction (all pFDR > 0.34), nor in static balance (BESS) or flexibility measures. This specificity of training effects suggests that 8 weeks of either core or mobility training provides sufficient stimulus for neuromuscular control adaptations but insufficient load or specificity for measurable improvements in explosive strength and power. This lack of improvement may be attributed to several interrelated factors, including the duration, intensity, and specificity of the intervention. Static balance is a multifaceted neuromuscular ability that involves the integration of sensory input from the visual, vestibular, and proprioceptive systems. Evidence suggests that improving balance, especially under static conditions, may require either a longer intervention period or training protocols specifically designed to challenge the postural control system. For example, Lesinski et al. performed a meta-analysis on balance training and highlighted that balance improvements are strongly moderated by the duration and intensity of the training, with interventions lasting between 11 and 12 weeks showing significantly larger effects on balance performance [69]. Thus, the 8 weeks of training in our study were probably not enough. Furthermore, BESS may not be sensitive enough to detect subtle changes over short timeframes, particularly in healthy individuals or athletic populations with already well-developed postural abilities. In this context, dynamic balance assessments, such as the YBT may offer greater sensitivity to neuromuscular adaptations induced by general training protocols like those mentioned previously. Improvements in tests such as SRT and Back Scratch are often more evident when the intervention includes regular and targeted stretching routines. According to Samson et al., flexibility (Sit-and-Reach performance) gains are best elicited through static stretching protocols lasting at least 30 s per muscle group, repeated 3 times, compared to the dynamic protocols [70]. If the intervention in question lacked dedicated stretching components or was of insufficient duration, it is unsurprising that no significant gains were detected. Similarly, horizontal jumping performance (hop tests) requires high levels of muscular strength, neuromuscular coordination, and explosive power. Significant improvements in such tests typically follow plyometric training regimens specifically tailored to the lower extremities. Markovic et al. underscore that plyometric training induces marked improvements in hopping ability, but only after sport-specific interventions [71]. Indeed, according to the principle of training specificity [72], athletes whose performance depends on generating force in the horizontal direction, such as sprinters and long jumpers, typically focus on plyometric training. In contrast, sports like basketball and volleyball demand vertical force production, primarily engaging in vertical jumping exercises. In the absence of such targeted exercises, global training interventions may not yield measurable changes in short-term hop performance. Moreover, plyometric exercise on an elastic surface has been shown to acutely decrease reactive power while maintaining leg stiffness [73], highlighting modality-specific adaptations of the stretch-shortening cycle.

Despite the meaningful findings, this study has limitations that must be acknowledged when interpreting the results. First and foremost, the absence of a true control group limits the ability to attribute observed improvements solely to the interventions applied. Including a control group in future studies would provide a more robust framework for causal inference. Secondly, the limited sample size, and the considerable inter-individual variability in responses, could indicate that the study may have been underpowered to reliably detect more subtle, modality-dependent effects even if supported by an a priori sample size calculation (*n* = 34 participants), the final analyzed sample (31 participants) was lower than the a priori sample size calculation required to adequately power the detection of a group-by-time interaction. This reduction in statistical power could increase the uncertainty of our null findings and limit definitive conclusions regarding the equivalence of the two interventions. For a transparent discussion of statistical power, between-group effect sizes (Cohen’s d) with 95% confidence intervals were calculated for all outcomes. In addition, as demonstrated by comparable studies [5] in a highly similar population (20 basketball players, age: 15.70 ± 0.75 years) and intervention (core stability training group or the traditional strength training group), sample sizes around *n* = 30 are often sufficient to detect significant effects in this research domain. Our use of hierarchical FDR correction, while methodologically rigorous, may have been overly conservative for detecting subtle between-group differences. However, the complete absence of even nominal time × group interactions (all *p* > 0.24) across all outcome measures suggests that any undetected differential effects would likely be small and of limited practical significance. However, a small cohort raises concerns about the representativeness of the findings, particularly in populations as heterogeneous as youth athletes, where biological age, training history, and sport specialization may vary significantly. Larger, adequately powered studies are needed to confirm the findings and establish normative data across different sports and age groups. Another limitation is the short duration of the intervention and the lack of long-term follow-up. Without post-intervention monitoring over time, it is unclear whether the improvements observed are transient adaptations or reflect durable neuromuscular changes. Incorporating longitudinal designs and delayed retention testing in future research would help clarify the sustainability of training effects and their potential role in long-term athletic development.

## 5. Conclusions

Based on the consistent lack of significant time × group interactions across all statistical methods and the application of rigorous multiple comparisons correction, we conclude that both training programs were similarly effective at improving dynamic balance and functional movement quality in young basketball players. Therefore, the choice between these two training modalities can be based on practical considerations rather than presumed efficacy differences, such as athlete preference, equipment availability, or their specific integration into the overall training schedule. This study aims to inform coaches, strength and conditioning professionals, and sports scientists on the relative effectiveness of adding core and mobility interventions, thereby helping optimize training protocols for performance enhancement and injury prevention in youth basketball. By focusing on adolescents, a population in a crucial stage of motor development and injury vulnerability, this work contributes to the body of evidence supporting early intervention with functional training modalities. Future studies should explore combined interventions using a larger sample, a longer training period, and standardized outcomes to find possible differences between the effects of the two training modalities and improve comparability and better define their role in sports performance enhancement.

## Figures and Tables

**Table 1:** Participants’ characteristics at the baseline were divided by group.

Variables	Mobility Training (M ± SD)	Core Training (M ± SD)	T Student’s	*p*-Value
age (years)	14.71 ± 2.27	14.88 ± 1.90	0.2890	0.824
height (cm)	174.73 ± 7.44	174.87 ± 10.29	−0.0381	0.970
weight (kg)	71.09 ± 10.99	69.14 ± 12.81	0.4012	0.692
BMI (kg/m^2^)	23.17 ± 2.30	22.63 ± 3.60	0.4388	0.665
basketball experience (years)	4.82 ± 2.71	3.80 ± 2.14	1.0695	0.295

Mean (M) and standard deviation (SD).

**Table 2:** Descriptive statistics (means and standard errors) for all outcome measures by group and time point.

Variable	Groups	Time	Mean	Std. Error	IC 95% (Lower)	IC 95% (Upper)
DYBT ANT (%)	CTG	T0	84.178	2.985	78.072	90.283
	CTG	T1	82.381	2.662	76.936	87.826
	MTG	T0	80.752	3.290	74.024	87.480
	MTG	T1	79.890	2.934	73.890	85.890
DYBT PL (%)	CTG	T0	95.295	2.218	90.760	99.831
	CTG	T1	103.403	3.627	95.985	110.821
	MTG	T0	91.406	2.444	86.408	96.405
	MTG	T1	106.640	3.997	98.466	114.814
DYBT PM (%)	CTG	T0	86.124	3.081	79.823	92.426
	CTG	T1	103.403	3.627	95.985	110.821
	MTG	T0	84.590	3.395	77.646	91.534
	MTG	T1	106.640	3.997	98.466	114.814
NDYBT ANT (%)	CTG	T0	83.430	3.649	75.966	90.893
	CTG	T1	81.970	3.194	75.438	88.502
	MTG	T0	78.544	4.021	70.320	86.768
	MTG	T1	81.732	3.519	74.534	88.930
NDYBT PL (%)	CTG	T0	98.161	2.199	93.664	102.659
	CTG	T1	107.271	3.203	100.720	113.822
	MTG	T0	90.142	2.423	85.186	95.098
	MTG	T1	104.040	3.530	96.821	111.259
NDYBT PM (%)	CTG	T0	87.210	2.744	81.598	92.821
	CTG	T1	96.519	4.022	88.294	104.744
	MTG	T0	88.167	3.023	81.984	94.351
	MTG	T1	101.781	4.432	92.717	110.845
BESS	CTG	T0	2.882	0.172	2.530	3.235
	CTG	T1	2.765	0.197	2.363	3.167
	MTG	T0	3.286	0.190	2.898	3.674
	MTG	T1	3.000	0.217	2.557	3.443
OST Total Score	CTG	T0	2.647	0.358	1.914	3.380
	CTG	T1	5.706	0.338	5.014	6.398
	MTG	T0	1.429	0.395	0.621	2.236
	MTG	T1	5.071	0.373	4.309	5.834
D Back Scratch (cm)	CTG	T0	7.447	1.596	4.182	10.712
	CTG	T1	8.324	1.750	4.744	11.903
	MTG	T0	8.079	1.759	4.481	11.676
	MTG	T1	7.350	1.929	3.405	11.295
ND Back Scratch (cm)	CTG	T0	1.694	2.360	−3.140	6.528
	CTG	T1	3.381	2.112	−0.945	7.707
	MTG	T0	0.964	2.523	−4.204	6.132
	MTG	T1	2.643	2.258	−1.982	7.268
Sit-and-Reach (cm)	CTG	T0	4.153	1.958	0.149	8.157
	CTG	T1	4.088	1.707	0.597	7.580
	MTG	T0	−1.236	2.157	−5.648	3.176
	MTG	T1	1.421	1.881	−2.426	5.269
Agility T-Test (s)	CTG	T0	14.192	0.421	13.331	15.052
	CTG	T1	13.619	0.349	12.905	14.334
	MTG	T0	14.520	0.464	13.572	15.468
	MTG	T1	13.824	0.385	13.036	14.611
D Hop Single Leg (cm)	CTG	T0	113.529	6.909	99.398	127.661
	CTG	T1	121.765	8.109	105.181	138.349
	MTG	T0	111.571	7.614	96.000	127.143
	MTG	T1	113.214	8.935	94.939	131.489
ND Hop Single Leg (cm)	CTG	T0	115.471	7.342	100.454	130.487
	CTG	T1	123.765	7.586	108.250	139.279
	MTG	T0	114.143	8.091	97.596	130.690
	MTG	T1	115.500	8.359	98.404	132.596
D Hop Triple (cm)	CTG	T0	402.706	21.908	357.898	447.513
	CTG	T1	399.176	20.975	356.277	442.076
	MTG	T0	362.500	24.142	313.125	411.875
	MTG	T1	364.714	23.114	317.442	411.987
ND Hop Triple (cm)	CTG	T0	395.941	19.042	356.996	434.886
	CTG	T1	407.176	18.452	369.438	444.915
	MTG	T0	362.643	20.983	319.728	405.558
	MTG	T1	366.571	20.333	324.985	408.157
D Crossover Triple (cm)	CTG	T0	318.412	24.574	268.152	368.672
	CTG	T1	322.235	25.035	271.034	373.437
	MTG	T0	294.000	27.079	238.616	349.384
	MTG	T1	307.786	27.587	251.364	364.207
ND Crossover Triple (cm)	CTG	T0	322.176	19.693	281.900	362.453
	CTG	T1	345.176	21.418	301.372	388.981
	MTG	T0	297.286	21.700	252.904	341.668
	MTG	T1	311.214	23.601	262.944	359.484
D Hop Test 6 m (s)	CTG	T0	3.089	0.104	2.877	3.302
	CTG	T1	2.999	0.107	2.780	3.217
	MTG	T0	3.184	0.114	2.950	3.418
	MTG	T1	3.071	0.118	2.830	3.311
ND Hop Test 6 m (s)	CTG	T0	3.022	0.116	2.785	3.258
	CTG	T1	2.999	0.107	2.780	3.217
	MTG	T0	3.208	0.127	2.947	3.469
	MTG	T1	3.071	0.118	2.830	3.311

Core Training Group (CTG); Mobility Training Group (MTG); baseline (T0); post-intervention (T1); dominant (D); non-dominant (ND); Y-Balance Test (YBT); anterior (ANT); posterolateral (PL); posteromedial (PM); Balance Error Scoring System (BESS); Overhead Squat Test (OST). Data are presented as estimated marginal means ± standard error, with the corresponding 95% confidence interval (CI). The CTG included *n* = 17 participants; the MTG included *n* = 14 participants.

## Data Availability

Data available in a publicly accessible repository: The original data presented in the study are openly available at https://github.com/ccortis/DatayouthBB.git (accessed on 20 October 2025).

## Supplementary Materials

The following supporting information can be downloaded at: https://www.mdpi.com/article/10.3390/sports13110398/s1, Table S1: Session completion and adherence rates for each participant (*n* = 31). Adherence was calculated as the percentage of completed sessions out of 24 scheduled sessions; Table S2: Homogeneity of Variances (Levene’s Test) for All Outcome Measures at T0 and T1; Table S3: Results for the Group × Time Interaction and Main Effect of Time from Repeated Measures ANOVA; Table S4: Main Effect of Time Across Statistical Methods: Linear Mixed Models (LMM) and ANOVA with False Discovery Rate (FDR) Correction; Table S5: Within-subjects analysis. Pre- to Post-Intervention (T0–T1) Change Scores and Effect Sizes (Hedges’ g_av) for Outcome Measures in the CTG and MTG Groups; Table S6: Between-Group Comparisons (CTG vs. MTG) for All Outcome Measures at Baseline (T0) and post-intervention (T1).

## Abbreviations

The following abbreviations are used in this manuscript:

CTG	core training group
MTG	mobility training group
BESS	balance error scoring system
YBT	y-balance test
OST	Overhead Squat test
SRT	Sit-and-Reach test
ANT	anterior reach
PL	posterolateral reach
PM	posteromedial reach
D	dominant limb
ND	non-dominant limb
M	mean
SD	standard deviation
MD	mean difference
NHE	Nordic hamstrings exercise
LSI	Limb Symmetry Index
FDR	False Discovery Rate
