# Supplementary material for: Comparison of the Core Training and Mobility Training Effects on Basketball Athletic Performance in Young Players: A Comparative Experimental Study
Source: Sports (Basel). 2025 Nov 6;13(11):398. doi: 10.3390/sports13110398 (PMC12655985; doi:10.3390/sports13110398)
Supplement: Supplementary file 1 [file sports-13-00398-s001.zip › sports-3881362-Table S1.pdf]

Table S1. Session completion and adherence rates for each participant (N = 31). Adherence was calculated as the percentage of completed sessions out of 24 scheduled sessions.

Participant ID	Scheduled sessions	Completed sessions	Adherence (%)
1	24	22	91.67
2	24	22	91.67
3	24	22	91.67
4	24	22	91.67
5	24	22	91.67
6	24	19	79.17
7	24	22	91.67
8	24	22	91.67
9	24	23	95.83
10	24	22	91.67
11	24	15	62.50
12	24	23	95.83
13	24	21	87.50
14	24	16	66.67
15	24	20	83.33
16	24	22	91.67
17	24	24	100.00
18	24	22	91.67
19	24	19	79.17
20	24	17	70.83
21	24	23	95.83
22	24	20	83.33
23	24	16	66.67
24	24	21	87.50
25	24	22	91.67
26	24	21	87.50
27	24	18	75.00
28	24	23	95.83
29	24	23	95.83
30	24	19	79.17
31	24	24	100.00
