# Supplementary material for: Comparison of the Core Training and Mobility Training Effects on Basketball Athletic Performance in Young Players: A Comparative Experimental Study
Source: Sports (Basel). 2025 Nov 6;13(11):398. doi: 10.3390/sports13110398 (PMC12655985; doi:10.3390/sports13110398)
Supplement: Supplementary file 1 [file sports-13-00398-s001.zip › sports-3881362-Table S2.pdf]

Table S2. Homogeneity of Variances (Levene's Test) for All Outcome Measures at T0 and T1

Variable	Time	Levene Statistic	Sig. (p-value)
T0 D YBT ANT (%)	T0	2.317	0.139
T1 D YBT ANT (%)	T1	0.672	0.419
T0 D YBT PL (%)	T0	6.234	0.018*
T1 D YBT PL (%)	T1	2.221	0.147
T0 D YBT PM (%)	T0	4.177	0.050
T1 D YBT PM (%)	T1	2.221	0.147
T0 ND YBT ANT (%)	T0	4.542	0.042*
T1 ND YBT ANT (%)	T1	0.029	0.866
T0 ND YBT PL (%)	T0	2.593	0.118
T1 ND YBT PL (%)	T1	0.076	0.785
T0 ND YBT PM (%)	T0	1.624	0.213
T1 ND YBT PM (%)	T1	0.914	0.347
T0 BESS (score)	T0	0.019	0.892
T1 BESS (score)	T1	0.231	0.635
T0 OST (score)	T0	1.568	0.220
T1 OST	T1	0.009	0.924
T0 D Back scratch (cm)	T0	0.094	0.761
T1 D Back scratch (cm)	T1	0.357	0.555
T0 ND Back scratch (cm)	T0	0.061	0.807
T1 ND Back scratch (cm)	T1	0.110	0.743
T0 Sit & reach (cm)	T0	0.163	0.689
T1 Sit & reach (cm)	T1	0.000	0.993
T0 Agility T-Test (sec)	T0	0.148	0.703
T1 Agility T-Test (sec)	T1	0.003	0.956
T0 D Hop single leg (cm)	T0	1.042	0.316
T1 D Hop single leg (cm)	T1	0.176	0.678
T0 ND Hop single leg (cm)	T0	2.177	0.151
T1 ND Hop single leg (cm)	T1	0.149	0.702
T0 D Hop test triple (cm)	T0	1.980	0.170
T1 D Hop test triple (cm)	T1	0.088	0.768
T0 ND Hop test triple (cm)	T0	0.009	0.927
T1 ND Hop test triple (cm)	T1	0.161	0.692
T0 D Crossover triple (cm)	T0	0.309	0.583
T1 D Crossover triple (cm)	T1	0.117	0.735
T0 ND Crossover triple (cm)	T0	0.000	0.993
T1 ND Crossover triple (cm)	T1	0.396	0.534
T0 D hop test 6 m (sec)	T0	0.266	0.610
T1 D hop test 6 m (sec)	T1	0.507	0.482
T0 ND hop test 6 m (sec)	T0	0.368	0.549
T1 ND hop test 6 m (sec)	T1	0.507	0.482

A significant p-value (Sig. < 0.05) indicates a violation of the homogeneity of variances assumption* = significant at p < 0.05. Balance Error Scoring System (BESS); Y-Balance Test (YBT); Anterior Reach (ANT); Posterolateral Reach (PL); Posteromedial Reach (PM); dominant limb (D); non-dominant limb (ND); mean (M); standard deviation (SD); overhead squat test (OST); degree of freedom (df).
