# Supplementary material for: Comparison of the Core Training and Mobility Training Effects on Basketball Athletic Performance in Young Players: A Comparative Experimental Study
Source: Sports (Basel). 2025 Nov 6;13(11):398. doi: 10.3390/sports13110398 (PMC12655985; doi:10.3390/sports13110398)
Supplement: Supplementary file 1 [file sports-13-00398-s001.zip › sports-3881362-Table S3.pdf]

Table S3. Results for the Group × Time Interaction and Main Effect of Time from Repeated Measures ANOVA

Variable	Effect	Pillai's Trace	F	p	η^2p
**D YBT ANT (%)**	Time	0.009	0.277	0.603	0.009
	Time × Group	0.001	0.034	0.855	0.001
	Group	-	0.773	0.387	0.026
**D YBT PL (%)**	Time	0.386	18.237	<0.001*	0.386
	Time × Group	0.055	1.700	0.203	0.055
	Group	-	0.008	0.927	0.000
**D YBT PM (%)**	Time	0.550	35.403	<0.001*	0.550
	Time × Group	0.018	0.521	0.476	0.018
	Group	-	0.051	0.823	0.002
**ND YBT ANT (%)**	Time	0.003	0.080	0.779	0.003
	Time × Group	0.020	0.581	0.452	0.020
	Group	-	0.392	0.536	0.013
**ND YBT PL (%)**	Time	0.410	20.176	<0.001*	0.410
	Time × Group	0.029	0.874	0.358	0.029
	Group	-	3.116	0.088	0.097
**ND YBT PM (%)**	Time	0.361	16.386	<0.001*	0.361
	Time × Group	0.020	0.578	0.453	0.020
	Group	-	0.531	0.472	0.018
**BESS (score)**	Time	0.057	1.757	0.195	0.057
	Time × Group	0.010	0.305	0.585	0.010
	Group	-	1.943	0.174	0.063
**OST (score)**	Time	0.843	155.864	<0.001*	0.843
	Time × Group	0.039	1.184	0.286	0.039
	Group	-	4.359	0.046*	0.131
**D Back Scratch (cm)**	Time	0.000	0.009	0.924	0.000
	Time × Group	0.037	1.101	0.303	0.037
	Group	-	0.005	0.943	0.000
**ND Back Scratch (cm)**	Time	0.109	3.432	0.075	0.109
	Time × Group	0.000	0.000	0.996	0.000
	Group	-	0.054	0.817	0.002
**Sit & Reach (cm)**	Time	0.047	1.415	0.244	0.047
	Time × Group	0.051	1.559	0.222	0.051
	Group	-	2.582	0.119	0.082
**Agility T-Test (s)**	Time	0.295	12.134	0.002*	0.295
	Time × Group	0.004	0.116	0.736	0.004
	Group	-	0.238	0.629	0.008
**D Hop Single Leg (cm)**	Time	0.084	2.668	0.113	0.084

	Time × Group	0.039	1.188	0.285	0.039
	Group	-	0.237	0.630	0.008
**ND Hop Single Leg (cm)**	Time	0.089	2.842	0.103	0.089
	Time × Group	0.048	1.468	0.235	0.048
	Group	-	0.200	0.658	0.007
**D Hop Test Triple (cm)**	Time	0.000	0.005	0.945	0.000
	Time × Group	0.003	0.092	0.764	0.003
	Group	-	1.501	0.230	0.049
**ND Hop Test Triple (cm)**	Time	0.046	1.403	0.246	0.046
	Time × Group	0.011	0.326	0.573	0.011
	Group	-	1.852	0.184	0.060
**D Crossover Triple (cm)**	Time	0.030	0.902	0.350	0.030
	Time × Group	0.010	0.289	0.595	0.010
	Group	-	0.296	0.591	0.010
**ND Crossover Triple (cm)**	Time	0.113	3.698	0.064	0.113
	Time × Group	0.008	0.223	0.640	0.008
	Group	-	1.025	0.320	0.034
**D Hop Test 6m (s)**	Time	0.135	4.531	0.042*	0.135
	Time × Group	0.002	0.057	0.812	0.002
	Group	-	0.312	0.580	0.011
**ND Hop Test 6m (s)**	Time	0.054	1.668	0.207	0.054
	Time × Group	0.028	0.849	0.364	0.028
	Group	-	0.705	0.408	0.024

* = significant at $p < 0.05$. Balance Error Scoring System (BESS); Y-Balance Test (YBT); Anterior Reach (ANT); Posterolateral Reach (PL); Posteromedial Reach (PM); dominant limb (D); non-dominant limb (ND); overhead squat test (OST); sit and reach test (SRT); degree of freedom (df); p value (p); f value (f); partial eta square (η^2p).
