# Supplementary material for: Comparison of the Core Training and Mobility Training Effects on Basketball Athletic Performance in Young Players: A Comparative Experimental Study
Source: Sports (Basel). 2025 Nov 6;13(11):398. doi: 10.3390/sports13110398 (PMC12655985; doi:10.3390/sports13110398)
Supplement: Supplementary file 1 [file sports-13-00398-s001.zip › sports-3881362-Table S4 .pdf]

Table S4. Main Effect of Time Across Statistical Methods: Linear Mixed Models (LMM) and ANOVA with False Discovery Rate (FDR) Correction.

Test Family	Variable	LMM p-value	LMM pFDR	ANOVA p-value
Y-Balance Test	D YBT PL	<0.001	<0.001*	<0.001*
	ND YBT PL	<0.001	<0.001*	<0.001*
	ND YBT PM	0.002	0.002*	<0.001*
	D YBT PM	0.013	0.013*	0.001*
	D YBT ANT	0.629	0.629	0.603
	ND YBT ANT	0.853	0.853	0.779
Functional Hop Tests	D Hop single leg	0.488	0.819	0.113
	ND Hop single leg	0.492	0.819	0.103
	D Hop test triple	0.965	0.965	0.945
	ND Hop test triple	0.674	0.819	0.246
	D Crossover triple	0.738	0.819	0.35
	ND Crossover triple	0.362	0.819	0.064
	D hop test 6m	0.342	0.819	0.042* #
	ND hop test 6m	0.819	0.819	0.207
Individual Tests	OST	<0.001	<0.001	<0.001*
	Agility T-Test	0.109	0.109	0.002* #
	BESS	0.299	0.299	0.195
	Sit & Reach	0.529	0.529	0.244
	D Back Scratch	0.928	0.928	0.924
	ND Back Scratch	0.540	0.540	0.075

* = significant at $p < 0.05$; #= Methodological discrepancy between LMM and ANOVA. FDR correction applied separately within test families, " Y-Balance Test " and " Functional Hop Tests ". Time × Group Interaction: Not significant in all LMM analyses ($p > 0.24$)
