# Supplementary material for: Comparison of the Core Training and Mobility Training Effects on Basketball Athletic Performance in Young Players: A Comparative Experimental Study
Source: Sports (Basel). 2025 Nov 6;13(11):398. doi: 10.3390/sports13110398 (PMC12655985; doi:10.3390/sports13110398)
Supplement: Supplementary file 1 [file sports-13-00398-s001.zip › sports-3881362-Table S5.pdf]

Table S5. Within-subjects analysis. Pre- to Post-Intervention (T0–T1) Change Scores and Effect Sizes (Hedges' g_av) for Outcome Measures in the CTG and MTG Groups

Variable	Group	Time	Mean difference (T0-T1)	Std. Error	Sig.	Relative Change (%)	95% Intervallo di Confidenza	Hedges' g_av	Between-Group Difference in Change (MD [95% CI])
D YBT ANT (%)	CTG	T0-T1	1.796	3.396	0.601	-2.1%	[-5.150, 8.743]	0.15	0.935 [-2.583, 4.453]
	MTG	T0-T1	0.862	3.742	0.820	-1.1%	[-6.793, 8.516]	0.07	
D YBT PL (%)	CTG	T0-T1	-8.108*	3.673	0.035	+8.5%	[-15.620, -0.595]	0.63	7.126 [-3.409, 17.661]
	MTG	T0-T1	-15.234*	4.048	<0.001	+16.7%	[-23.512, -6.956]	1.08	
D YBT PM (%)	CTG	T0-T1	-17.279*	4.442	<0.001	+20.1%	[-26.364, -8.194]	1.12	4.771 [-8.176, 17.718]
	MTG	T0-T1	-22.050*	4.895	<0.001	+26.1%	[-32.061, -12.039]	1.30	
ND YBT ANT (%)	CTG	T0-T1	1.459	4.099	0.724	-1.7%	[-6.923, 9.842]	0.10	4.648 [0.056, 9.240]*
	MTG	T0-T1	-3.189	4.516	0.486	+4.1%	[-12.426, 6.049]	0.20	
ND YBT PL (%)	CTG	T0-T1	-9.110*	3.442	0.013	+9.3%	[-16.150, -2.070]	0.76	4.789 [-5.228, 14.806]
	MTG	T0-T1	-13.899*	3.793	<0.001	+15.4%	[-21.657, -6.141]	1.06	
ND YBT PM (%)	CTG	T0-T1	-9.309*	3.806	0.021	+10.7%	[-17.093, -1.526]	0.70	4.305 [-6.852, 15.462]
	MTG	T0-T1	-13.614*	4.194	0.003	+15.4%	[-22.190, -5.037]	0.93	
BESS (score)	CTG	T0-T1	0.118	0.205	0.570	-4.1%	[-0.301, 0.536]	0.16	-0.168 [-0.723, 0.387]
	MTG	T0-T1	0.286	0.225	0.215	-8.7%	[-0.175, 0.747]	0.36	
OST (score)	CTG	T0-T1	-3.059*	0.361	<0.001	+115.5%	[-3.797, -2.321]	2.44	0.584 [-0.539, 1.707]
	MTG	T0-T1	-3.643*	0.398	<0.001	+254.9%	[-4.456, -2.830]	2.63	
D Back scratch (cm)	CTG	T0-T1	-0.876	1.028	0.401	+11.8%	[-2.979, 1.226]	0.24	-1.606 [-4.239, 1.027]
	MTG	T0-T1	0.729	1.133	0.525	-9.0%	[-1.588, 3.045]	0.18	
ND Back scratch (cm)	CTG	T0-T1	-1.687	1.241	0.185	+99.6%	[-4.230, 0.855]	0.38	-0.008 [-5.219, 5.203]
	MTG	T0-T1	-1.679	1.327	0.216	+174.2%	[-4.397, 1.040]	0.36	
Sit & reach (cm)	CTG	T0-T1	0.065	1.465	0.965	-1.6%	[-2.931, 3.060]	0.01	2.722 [1.081, 4.363]*
	MTG	T0-T1	-2.657	1.614	0.111	+215.0%	[-5.958, 0.644]	0.47	
Agility-T Test (sec)	CTG	T0-T1	0.572*	0.245	0.026	-4.0%	[0.072, 1.073]	0.66	-0.124 [-0.869, 0.621]
	MTG	T0-T1	0.696*	0.270	0.015	-4.8%	[0.145, 1.248]	0.74	
D Hop single leg (cm)	CTG	T0-T1	-8.235	4.064	0.052	+7.3%	[-16.548, 0.077]	0.58	-6.593 [-21.773, 8.587]
	MTG	T0-T1	-1.643	4.479	0.716	+1.5%	[-10.803, 7.517]	0.10	
ND Hop single leg (cm)	CTG	T0-T1	-8.294*	3.847	0.040	+7.2%	[-16.162, -0.426]	0.61	-6.937 [-17.693, 3.819]
	MTG	T0-T1	-1.357	4.239	0.751	+1.2%	[-10.028, 7.313]	0.09	
D Hop test triple (cm)	CTG	T0-T1	3.529	12.729	0.784	-0.9%	[-22.504, 29.563]	0.08	5.744 [-17.947, 29.435]
	MTG	T0-T1	-2.214	14.027	0.876	+0.6%	[-30.902, 26.474]	0.04	
ND Hop test triple (cm)	CTG	T0-T1	-11.235	8.604	0.202	+2.8%	[-28.833, 6.363]	0.37	-7.307 [-35.497, 20.883]
	MTG	T0-T1	-3.929	9.482	0.682	+1.1%	[-23.321, 15.464]	0.12	
D Crossover triple (cm)	CTG	T0-T1	-3.824	12.462	0.761	+1.2%	[-29.310, 21.663]	0.08	9.963 [-11.833, 31.759]
	MTG	T0-T1	-13.786	13.732	0.324	+4.7%	[-41.871, 14.299]	0.29	
ND Crossover triple (cm)	CTG	T0-T1	-23.000	12.905	0.085	+7.1%	[-49.393, 3.393]	0.51	-9.072 [-45.231, 27.087]
	MTG	T0-T1	-13.929	14.220	0.335	+4.7%	[-43.013, 15.155]	0.28	
D hop test 6m (sec)	CTG	T0-T1	0.091	0.064	0.170	-2.9%	[-0.041, 0.222]	0.40	-0.023 [-0.175, 0.129]
	MTG	T0-T1	0.114	0.071	0.121	-3.5%	[-0.032, 0.259]	0.46	
ND hop test 6m (sec)	CTG	T0-T1	0.023	0.083	0.785	-0.8%	[-0.147, 0.193]	0.08	-0.114 [-0.295, 0.067]
	MTG	T0-T1	0.137	0.092	0.146	-4.3%	[-0.051, 0.325]	0.43	

Interpretation Criteria for Hedges' g_av: $|g| < 0.20$ = Negligible; $0.20 \leq |g| < 0.50$ = Small; $0.50 \leq |g| < 0.80$ = Medium; $|g| \geq 0.80$ = Large; $|g| \geq 2.00$ = Very Large. * = significant at $p < 0.05$; Hedges' g_av = Mean difference / SD pooled, bias-corrected; All tests corrected with Bonferroni method for multiple comparisons; Variables listed as T0 and T1; Negative values indicate a reduction from T0 and T1. relative change (%) for groups is calculated as (Mean T1 - Mean T0) / Mean T0 * 100; Between-Group Difference in Change is calculated as (Mean MTG T1 - Mean MTG T0) - (Mean CTG T1 - Mean CTG T0). Balance Error Scoring System (BESS); Y-Balance Test (YBT); Anterior Reach (ANT); Posterolateral Reach (PL); Posteromedial Reach (PM); dominant limb (D); non-dominant limb (ND); mean (M); standard deviation (SD); Overhead Squat Test (OST); control group (CTG); mobility group (MTG)
