# Supplementary material for: Comparison of the Core Training and Mobility Training Effects on Basketball Athletic Performance in Young Players: A Comparative Experimental Study
Source: Sports (Basel). 2025 Nov 6;13(11):398. doi: 10.3390/sports13110398 (PMC12655985; doi:10.3390/sports13110398)
Supplement: Supplementary file 1 [file sports-13-00398-s001.zip › sports-3881362-Table S6.pdf]

Table S6. Between-Group Comparisons (CTG vs. MTG) for All Outcome Measures at Baseline (T0) and post-intervention (T1)

	Variable	Mean difference (CTG - MTG)	Std Error	Sig. (p)	95% CIs	Effect Size (d)	95% CI for d
T0	D YBT ANT (%)	3.426	4.442	0.447	[-5.660, 12.511]	0.24	[-0.47, 0.95]
T1	D YBT ANT (%)	2.491	3.961	0.534	[-5.611, 10.593]	0.21	[-0.50, 0.92]
T0	D YBT PL (%)	3.889	3.300	0.248	[-2.860, 10.639]	0.32	[-0.39, 1.03]
T1	D YBT PL (%)	-3.237	5.397	0.553	[-14.276, 7.801]	-0.17	[-0.88, 0.54]
T0	D YBT PM (%)	1.534	4.585	0.740	[-7.842, 10.911]	0.10	[-0.61, 0.81]
T1	D YBT PM (%)	-3.237	5.397	0.553	[-14.276, 7.801]	-0.17	[-0.88, 0.54]
T0	ND YBT ANT (%)	4.886	5.430	0.376	[-6.220, 15.992]	0.27	[-0.44, 0.98]
T1	ND YBT ANT (%)	0.238	4.752	0.960	[-9.482, 9.958]	0.02	[-0.69, 0.73]
T0	ND YBT PL (%)	8.019*	3.272	0.021	[1.327, 14.712]	0.66	[-0.06, 1.38]
T1	ND YBT PL (%)	3.231	4.766	0.503	[-6.518, 12.979]	0.25	[-0.46, 0.96]
T0	ND YBT PM (%)	-0.957	4.083	0.816	[-9.307, 7.393]	-0.07	[-0.78, 0.64]
T1	ND YBT PM (%)	-5.262	5.984	0.386	[-17.501, 6.978]	-0.26	[-0.97, 0.45]
T0	BESS (score)	-0.403	0.256	0.126	[-0.927, 0.121]	-0.44	[-1.15, 0.28]
T1	BESS (score)	-0.235	0.293	0.428	[-0.834, 0.363]	-0.23	[-0.94, 0.48]
T0	OST (score)	1.218*	0.533	0.030	[0.128, 2.309]	0.63	[-0.09, 1.35]
T1	OST (score)	0.634	0.504	0.218	[-0.395, 1.664]	0.35	[-0.36, 1.06]
T0	D Backscratch (cm)	-0.632	2.375	0.792	[-5.489, 4.226]	-0.07	[-0.78, 0.64]
T1	D Backscratch (cm)	0.974	2.604	0.711	[-4.353, 6.300]	0.10	[-0.61, 0.81]
T0	ND Backscratch (cm)	0.729	3.455	0.834	[-6.347, 7.806]	0.06	[-0.65, 0.77]
T1	ND Backscratch (cm)	0.738	3.092	0.813	[-5.594, 7.071]	0.07	[-0.64, 0.78]
T0	Sit & reach (cm)	5.389	2.913	0.075	[-0.569, 11.346]	0.51	[-0.21, 1.22]
T1	Sit & reach (cm)	2.667	2.540	0.302	[-2.529, 7.862]	0.29	[-0.42, 1.00]
T0	Agility T Test (sec)	-0.328	0.626	0.604	[-1.608, 0.952]	-0.14	[-0.85, 0.57]
T1	Agility T Test (sec)	-0.204	0.520	0.697	[-1.267, 0.859]	-0.11	[-0.82, 0.60]
T0	D Hop singleleg (cm)	1.958	10.282	0.850	[-19.070, 22.986]	0.05	[-0.66, 0.76]
T1	D Hop singleleg (cm)	8.550	12.066	0.484	[-16.128, 33.228]	0.20	[-0.51, 0.91]
T0	ND Hop singleleg (cm)	1.328	10.925	0.904	[-21.017, 23.673]	0.03	[-0.68, 0.74]
T1	ND Hop singleleg (cm)	8.265	11.288	0.470	[-14.822, 31.351]	0.20	[-0.51, 0.91]
T0	D Hoptest triple (cm)	40.206	32.601	0.227	[-26.470, 106.882]	0.34	[-0.37, 1.05]
T1	D Hoptest triple (cm)	34.462	31.212	0.279	[-29.374, 98.298]	0.31	[-0.40, 1.02]
T0	ND Hoptest triple (cm)	33.298	28.335	0.249	[-24.654, 91.250]	0.33	[-0.38, 1.04]
T1	ND Hoptest triple (cm)	40.605	27.458	0.150	[-15.552, 96.762]	0.41	[-0.30, 1.12]
T0	D Crossover triple (cm)	24.412	36.568	0.510	[-50.377, 99.201]	0.19	[-0.52, 0.90]
T1	D Crossover triple (cm)	14.450	37.253	0.701	[-61.741, 90.640]	0.11	[-0.60, 0.82]
T0	ND Crossover triple (cm)	24.891	29.304	0.403	[-35.042, 84.824]	0.24	[-0.47, 0.95]
T1	ND Crossover triple (cm)	33.962	31.871	0.295	[-31.221, 99.145]	0.30	[-0.41, 1.01]
T0	D hoptest 6m (sec)	-0.095	0.154	0.544	[-0.411, 0.221]	-0.17	[-0.88, 0.54]
T1	D hoptest 6m (sec)	-0.072	0.159	0.654	[-0.397, 0.253]	-0.13	[-0.84, 0.58]
T0	ND hoptest 6m (sec)	-0.186	0.172	0.289	[-0.538, 0.166]	-0.30	[-1.01, 0.41]
T1	ND hoptest 6m (sec)	-0.072	0.159	0.654	[-0.397, 0.253]	-0.13	[-0.84, 0.58]

Data presented as Mean Difference (CTG – MTG) with standard error, p-value, and 95% confidence intervals (95% CIs). A positive mean difference indicates a higher value in the CTG group compared to the MTG group. An asterisk (*) denotes a statistically significant between-group difference ($p < 0.05$). Balance Error Scoring System (BESS); Y-Balance Test (YBT); Anterior Reach (ANT); Posterolateral Reach (PL); Posteromedial Reach (PM); dominant limb (D); non-dominant limb (ND); mean (M); standard deviation (SD); Overhead Squat Test (OST); control group (CTG); mobility group (MTG).
